# Real-Time Decision Fusion for Multimodal Neural Prosthetic Devices

**DOI:** 10.1371/journal.pone.0009493

**Published:** 2010-03-02

**Authors:** James Robert White, Todd Levy, William Bishop, James D. Beaty

**Affiliations:** 1 Applied Mathematics and Scientific Computation Program, University of Maryland – College Park, College Park, Maryland, United States of America; 2 The Johns Hopkins University Applied Physics Laboratory, Laurel, Maryland, United States of America; George Mason University, United States of America

## Abstract

**Background:**

The field of neural prosthetics aims to develop prosthetic limbs with a brain-computer interface (BCI) through which neural activity is decoded into movements. A natural extension of current research is the incorporation of neural activity from multiple modalities to more accurately estimate the user's intent. The challenge remains how to appropriately combine this information in real-time for a neural prosthetic device.

**Methodology/Principal Findings:**

Here we propose a framework based on *decision fusion*, i.e., fusing predictions from several single-modality decoders to produce a more accurate device state estimate. We examine two algorithms for continuous variable decision fusion: the Kalman filter and artificial neural networks (ANNs). Using simulated cortical neural spike signals, we implemented several successful individual neural decoding algorithms, and tested the capabilities of each fusion method in the context of decoding 2-dimensional endpoint trajectories of a neural prosthetic arm. Extensively testing these methods on random trajectories, we find that on average both the Kalman filter and ANNs successfully fuse the individual decoder estimates to produce more accurate predictions.

**Conclusions:**

Our results reveal that a fusion-based approach has the potential to improve prediction accuracy over individual decoders of varying quality, and we hope that this work will encourage multimodal neural prosthetics experiments in the future.

## Introduction

Each year ∼150,000 people in the United States undergo an arm or leg amputation [1]. An estimated 1.7 million amputees live in the United States [2] and millions more throughout the world. Reasons for limb loss range from physical trauma to infection to diseases such as diabetes and cancer. Regardless of the cause, the loss of a limb dramatically affects a person's life, making many simple tasks unbearably difficult. Over the past decade, prosthetic limbs have been developed to incorporate electrical signals from indirect muscles for user control – this is known as conventional prosthetic control. The emerging field of neural prosthetics goes further, interpreting the neural activity of the user for more intuitive control of prosthetic devices.

The problem of translating neural activity into direct movements is known as *neural decoding*. Types of recorded neural activity that can be decoded include cortical single-neuron action potentials (spikes) [3], [4], local field potentials (LFPs) [5], [6], [7], and activity on the surface of the brain via electrocorticography (ECoG) [8], [9], [10], [11], [12], electromyography (EMG) [13], or electroencephalography (EEG) [14], [15], [16]. Each of these modalities offers particular advantages and limitations. For example, the surface-based EEG and ECoG recording platforms are relatively non-invasive, but provide poor spatial resolution (millimeters or centimeters). In contrast, spike signals provide accurate firing rates of single neurons, but this modality is highly invasive and prone to electrode failure [17], [18], [19]. While spike decoding is useful for predicting prosthetic endpoint trajectories, recent studies have demonstrated that modalities with less resolution are superior at encoding more general movement regimes [20], [21].

Each modality involves specific hardware (e.g. electrodes), and analysis of these signals requires algorithms carefully designed to predict the user's intent given the characteristics of the signal (e.g. signal-to-noise ratio, noise distributions, dependencies). Neural decoding algorithms generate a *state estimate* as either a discrete classification (e.g. a gating classifier results in a decision for movement or no movement [22]) or a prediction of continuous variables (e.g. three-dimensional position and velocity estimates for the endpoint of a limb [23]). Moreover, some algorithms calculate confidence regions for state estimates, thereby providing additional information for the robotic controls interface.

Decoding of individual neural modalities is a consistently improving field with many robust methodologies. However, due to the limitations of current recording technologies, more advanced prosthetic limbs will require multiple neural signals with varying information content in order to achieve full functionality. A major computational challenge is to analyze all signals simultaneously to provide the best estimate of the user's desired movement.

Here we present a framework for combining information from multiple modalities to more accurately decode user intent for a prosthetic device. There are two solution paradigms for this problem: *data* fusion and *decision* fusion. Data fusion (low-level fusion) merges several raw signals prior to analysis, while decision fusion (high-level fusion) acts as a post-processor to merge the results of individual data analyses. Fusion frameworks have been shown to improve prediction accuracy in a wide range of fields including biometric identity confirmation [24], [25], [26], surface-to-air defense [27], robot navigation [28], [29], [30], [31], image segmentation [32], and diagnosis of disease [33], [34].

Though data fusion allows for all information to be assessed at once by a single algorithm, current hardware architectures for neural prostheses are parallelized with multiple recording platforms and processors, inherently advocating parallelized decoding prior to a final state prediction. As most decoding algorithms are optimized for specific modalities, we employ techniques for *decision fusion*, where we incorporate the estimates from each individual decoder into a single device state estimate.

In this report, we examine two algorithms for decision fusion of continuous variables: the Kalman filter and artificial neural networks (ANNs). We implemented three of the most successful individual neural decoding algorithms with simulated cortical neural spike data to test the capabilities of each fusion method. Through these simulations, we reveal the advantages and limitations of these approaches. Our methodology provides a flexible framework for fusing state estimates from decoding algorithms with different properties and hopefully will encourage multimodal experiments for improved control of sophisticated neural prosthetic devices.

## Materials and Methods

### The Kalman Filter for Decision Fusion

We first formulate decision fusion in terms of Bayesian statistical inference. For our purposes, measurements are predictions from the individual decoders, and the system state is the 2-dimensional velocity vector of the prosthetic endpoint. Given the history of all measurements up to timestep *k*, 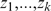, we seek to find the most likely state of the system, 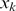, which is equivalent to the mode of the *posterior* probability distribution:
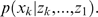
The Kalman filter is a well-known recursive Bayesian algorithm for solving this problem. This algorithm efficiently solves for the mode of the system posterior at time *k* given the set of all measurements of the system through time *k*. The Kalman filter first assumes a linear-Gaussian relationship between the current state of the system and the state at the previous timestep:
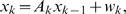


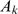
 is a coefficient matrix, and 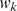 is a Gaussian error term with mean 0 and covariance matrix 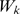. The Kalman filter further assumes a linear-Gaussian relationship between the measurements and the state of the system at each timestep:
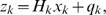


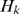
 is a coefficient matrix, and 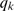 is a Gaussian error term with mean 0 and covariance 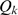. Under these assumptions the Kalman filter provides an “optimal” estimate of the state posterior minimizing the mean-squared error.

To simplify the model, we assume 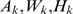 and 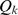 are time-invariant, and so closed-form maximum joint probability solutions exist for each matrix [35]:
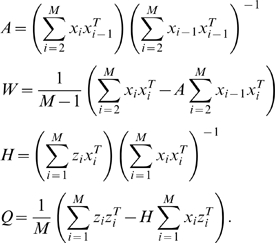
See [36] for an excellent review of Kalman filter theory.

### Artificial Neural Networks for Decision Fusion

**Figure 1:**
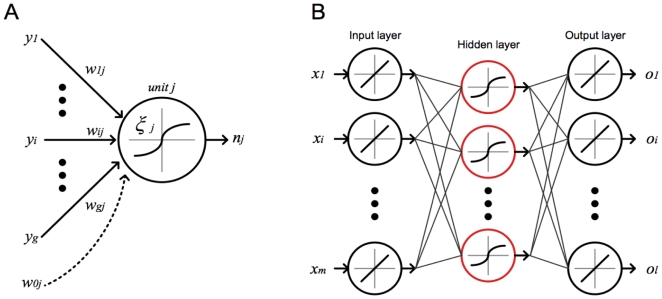
Conceptual design of an artificial neural network. (**A**) Each individual unit in the network accepts a weighted sum of input values, producing a single net activation value, *n_j_*. (**B**) A three-layer network topology. This topology is *feed-forward* and *fully-connected*, that is, each unit links to all units in the layer directly after it.

Artificial neural networks have also been used as a method for fusing decisions from supervised classifiers and data from multiple sensors. An ANN is a mathematical model composed of simulated neuron *units* and links between units. Each unit has a corresponding activation function, *ξ*, that accepts a weighted sum of input values and outputs a net activation value. Activation functions may be piecewise constant, linear, or nonlinear. The general form of the net activation value for unit *j* is:
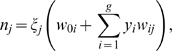
where 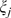 is the activation function of the *j^th^* unit, 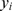 is the net activation from unit *i*, and 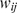 is the weight from unit *i* into unit *j* (see Figure 1).

We implemented feed-forward ANNs with either one or two hidden layers. At each timestep, the state estimates of each individual decoder are provided to the input units, while the output layer produces a fused estimate of the x and y velocities. The activation functions for all hidden units are tansigmoid, and the output layer uses linear functions. To train each ANN, we employed the scaled conjugate gradient method for learning the neuron weights and the mean squared error as a criterion function. We additionally optimized the number of hidden units by searching the space of all permutations ranging from one to 12 hidden units in the first layer, and zero to 11 hidden units in the second layer. Thus, 144 ANNs were examined to find an optimal selection of hidden units within each layer.

### Simulated Neural Data

Similar to Moran and Schwartz [37] and Wu *et al.* [35] we model neuron spiking activity according to a cosine-tuning function relating the “preferred direction” of each neuron to the direction and velocity of an endpoint. Thus, the firing rate of a neuron at time *t* follows a Poisson distribution with mean *z_t_*:
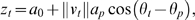
where *θ_p_* is the preferred direction of the neuron, and *θ_t_* and *v_t_* are the angle and velocity of the movement, respectively. All experiments modeled 50 input neurons. Simulated neurons were randomly assigned preferred directions (within range [−*π*, *π*]), and parameters *a_0_* and *a_p_* varied for each experiment.

### Individual Decoder Algorithms

#### Kalman filter

The Kalman filter framework as a single neural decoder was very similar to that of the fusion implementation. The individual Kalman filter modeled the relationship between neural spikes and the state of the device as a linear Gaussian process. The dimensionality of this observation model was larger than the observation model used for the fusion Kalman filter.

#### A variant of the population vector algorithm

We employed a model similar to the population vector algorithm (PVA) described in Moran and Schwartz [37] to decode the intended endpoint velocities. The equation used to generate our simulated neural data is described above, and the population vector algorithm utilizes the following model:
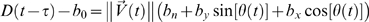
In PVA, *τ*, *b_0_*, *b_n_*, *b_y_*, and *b_x_* must be estimated before determining *θ* and ∥*V*(*t*)∥ whereas in our model we only needed to estimate *a_0_*, *a_p_*, and *θ_p_*. We can estimate these parameters using an iterative Taylor series approximation. As long as there are more neurons than the number of parameters (in this case 3), we can then estimate the angle and speed, or equivalently, the x and y components of the velocity.

#### Optimal linear decoder

The linear filters constructed for decoding used sliding windows of length four timepoints to form a response matrix of neuron firing rates. To train each filter, we performed a multiple regression of the x and y velocities over a response matrix spanning the entire training set:
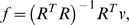
where *f* is the linear filter, *R* is the response matrix, and *v* is a vector containing the x or y velocities. For any response matrix, *R*, the linear prediction is:
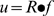
Note that for this filter, there exists a delay the same length as the window size, and we translated each decoded trajectory accordingly.

### Decision Fusion Evaluation

**Figure 2:**
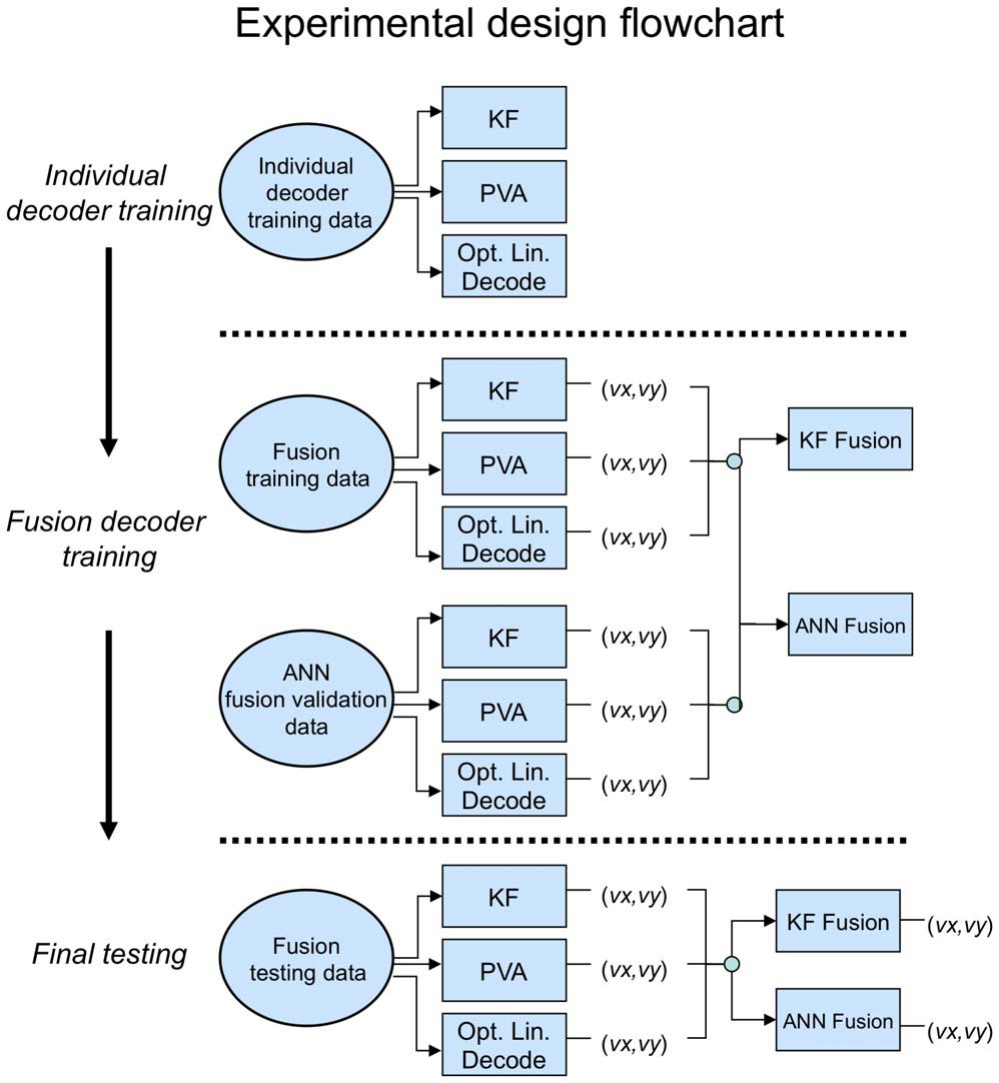
Experimental design for fusion trials. Flowchart describing fusion of Kalman filter (KF), PVA, and the optimal linear decodes using the Kalman filter and ANNs. Experimental trials contained three major phases: (i) individual decoder training, (ii) fusion decoder training, and (iii) final testing. In each experiment, individual decoders were first trained using the same simulated spike count data. Next, fusion decoders were trained on the individual decoders' outputs (predicted velocity components in *x* and *y* dimensions) for a separate **fusion training** dataset. An additional **validation** dataset was employed to prevent overtraining of ANNs. In final testing, trained individual decoders were used to predict the 2-d velocities, which were then compiled as input for fusion decoders. Endpoint velocity predictions from all decoders were then compared for accuracy. See Methods for details of the evaluation methodology.

Evaluation trials were designed to compare the accuracy of individual decoder predictions to “fused” results obtained from the Kalman filter and ANNs. Below we describe the three major components of each experiment: (i) individual decoder training, (ii) fusion decoder training, and (iii) final testing. See Figure 2 for a graphical description.

#### Individual decoder training

Each single decoder (PVA, Kalman filter, and optimal linear decoder) was trained on an identical dataset composed of 50 simulated neuron spike observations with a corresponding endpoint path. Trials associated with high-quality and poor-quality decoders used training datasets with 3,000 and 1,500 time-steps, respectively.

#### Fusion decoder training

When training the decision fusion algorithms, a set of predictions for each individual decoder is required. One could simply let the single decoders make predictions based on the initial training dataset, but this could lead to overfitting and poor performance on new data. To avoid this, a second dataset for *fusion training* was generated separately for the decision fusion algorithms. This dataset uses the same 50 simulated neurons, but for a different endpoint trajectory of 10,000 timesteps. Trained individual decoders were used to predict the two-dimensional endpoint velocity of the limb based on the fusion training dataset. At each timepoint, the predictions (*v_x_*, *v_y_*) were formed into an observation vector, (3 individual decoders ×2 velocity components = 6 components to each observation vector). The set of all observation vectors were used as a training set for the fusion Kalman filter and ANNs. To prevent overfitting the ANNs, a secondary *ANN fusion validation* dataset for a limited trajectory (3,000 timesteps) was employed in the same manner as the fusion training dataset.

#### Final testing

After training the fusion and individual decoders, a set of trajectories and corresponding spike signals were generated for testing. Each trajectory represented 3,000 timesteps. For each trial, cortical spikes counts were input to individual decoders, which output predictions for *x* and *y* velocity estimates. Endpoint velocity predictions were then compiled into observation vectors and fed to the fusion algorithms for final predictions. Predictions from the individual decoders and the fusion methods were finally compared to the true endpoint velocities using root mean squared error.

### Random Trajectory Generation

We generated random trajectories in 2-dimensional position space according to the following model:
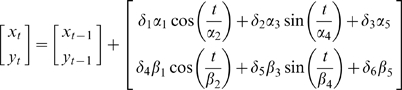
The parameters of the model for each trajectory were chosen by sampling from the following statistical distributions:
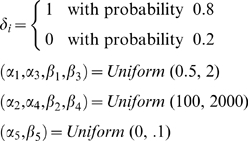
The space of possible trajectories spanned both nonlinear and linear relationships.

## Results

We present the fusion problem in the context of estimating the endpoint velocity of a prosthetic arm using several different decoding algorithms of varying accuracy. Decoding studies often focus on endpoint trajectories, leaving the controls of the limb to determine optimal joint positions and velocities by inverse kinematics.

### Simulated Fusion Trials

To investigate these fusion methods, we simulated neural spike data and implemented the following algorithms for spike decoding: standard Kalman filter [35], [38], [39], optimal linear filter [40], [41], and a variant of the population vector algorithm (PVA) [23], [42], [43], [44]. The optimal linear filter uses a sliding window to look back in time to estimate the current state of the arm using a multidimensional linear regression. A separate linear filter is developed for each variable of interest (in our case, x and y velocities). The population vector algorithm predicts velocity and direction using the “preferred direction” of each neuron in conjunction with a model relating neural activity to speed and direction of movement. We simulate single-neuron spike firing rates as a function of the velocity and direction of the limb in x and y coordinates. All simulated neuron firing rates were perfectly cosine-tuned and included Poisson noise (see Methods for detailed descriptions of decoders and simulated firing rates).

### Initial Testing of Fusion Algorithms

Testing the fusion algorithms first required training each individual decoder. Each trained algorithm was then used to decode a fusion training dataset and a separate fusion validation dataset for training the artificial neural network. The use of a validation dataset prevents overtraining of the ANN. The outputs of the trained algorithms (in our case x and y velocities) served as inputs to train the fusion algorithms (Figure 2). All trained algorithms decoded velocities for four testing datasets. The four test sets were generated independently from previous training and validation data, and tested a range of trajectories from simple to complex.

**Figure 3:**
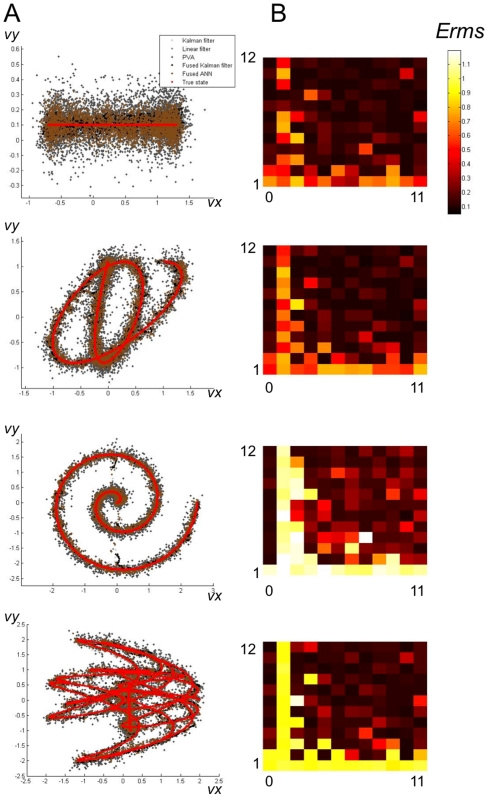
Initial testing of fusion decoders. (**A**) Decoded velocity trajectories for four trials. The true velocities are shown in red. The fused ANN and fused Kalman filter decodes are shown in brown and black, respectively. Individual decoders are plotted in varying shades of grey. (**B**) *E_rms_* of 144 neural networks for four trial decodes. We examined a range of single and double hidden-layer networks to optimize the fusion results. Rows correspond to 1st-layer sizes, while columns are 2^nd^-layer sizes. Note the first column in each matrix corresponds to all single hidden-layer networks. Interestingly, many single hidden-layer networks outperform more complex networks, indicating the dynamic accuracies of different neural network topologies. Table 2 displays the corresponding *E_rms_* values for each decoder.

We measure the accuracy of the decoded trajectories in terms of the root mean squared error (*E_rms_*) in velocity space. If 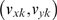 is the true velocity and 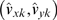 is the estimate 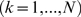, then:
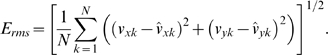

Figure 3B displays ANN *E_rms_* results of optimizing the number of neurons in each hidden layer for each of four trials. Note that the first column of cells in each matrix corresponds to a single hidden-layer network. We observe that neural networks with a single unit in the first or second hidden layer perform poorly. We also see that the single hidden-layer networks typically perform just as well as many of the double hidden-layer networks. This experiment reveals the dynamic nature of network accuracy depending on the topology employed. Indeed more complex networks do not necessarily provide the best performance. A notable example is the double hidden-layer network with nine and three units in the first and second hidden layers, respectively. The *E_rms_* for this network is relatively high (compared to its immediate neighbors) for trials 1 through 3, but this disappears for trial 4. ANN topologies with the lowest *E_rms_* were all different for each trial (Table 1). This suggests that optimizing the number of neurons is data dependent and no one topology will always result in the best performance.

**Table 1:** Artificial neural networks with lowest E_rms_ for each trial.

Trial	1	2	3	4
**No. units in 1^st^ hidden layer**	8	11	12	6
**No. units in 2^nd^ hidden layer**	11	7	10	10
***E_rms_*** **±** ***s.e.***	0.085±0.002	0.083±0.002	0.097±0.003	0.103±0.004

Searching the space of possible topologies seen in Figure 3B, the most accurate decoding ANNs had different topologies for each trial.

The final decoded trajectories are presented in Figure 3A. For each trial, the best performing ANN is plotted in brown. True velocities are plotted in red. **Table 2** shows the *E_rms_* for all individual decoders and fusion algorithms. In three out of four trials, the Kalman filter fusion resulted in the most accurate decode. In the remaining trial, the fused ANN decoded velocities had the lowest *E_rms_*. In all four trials, at least one fusion algorithm outperformed all three individual decoders. Furthermore, across individual decoders, no single method was consistently superior.

**Table 2:** *E_rms_* ± standard error (s.e.) for four trials.

Trial	1	2	3	4
**Kalman filter**	0.073±0.001	0.069±0.001	0.126±0.004	0.090±0.004
**Linear filter**	0.093±0.002	0.090±0.002	0.102±0.003	0.107±0.005
**Population vector**	0.174±0.003	0.172±0.003	0.179±0.003	0.203±0.011
**Kalman fusion**	**0.059±0.001**	**0.062±0.001**	0.119±0.004	**0.066±0.002**
**ANN fusion**	0.0850±0.002	0.083±0.002	**0.097±0.003**	0.103±0.004

Bold elements in tables have the lowest *E_rms_* for the trial. In all four trials, the fusion algorithms had more accurate results than at least two of the three individual decoders. In trials 1, 2, and 4, the Kalman fusion method produced the lowest *E_rms_*. In trial 3, the fused ANN decisions were the most accurate.

### Variable Decoding Accuracies

The accuracy of neural decoders depends not only on the sophistication of the decoding algorithms but also on the physical recording locations and the nature of the signals. A few millimeters of discrepancy in electrode placement can dramatically impact decoding accuracy [20]. Thus, in devices with multimodal recording, no one decoded modality is likely to provide superior performance over others for the full spectrum of functionality.

**Figure 4:**
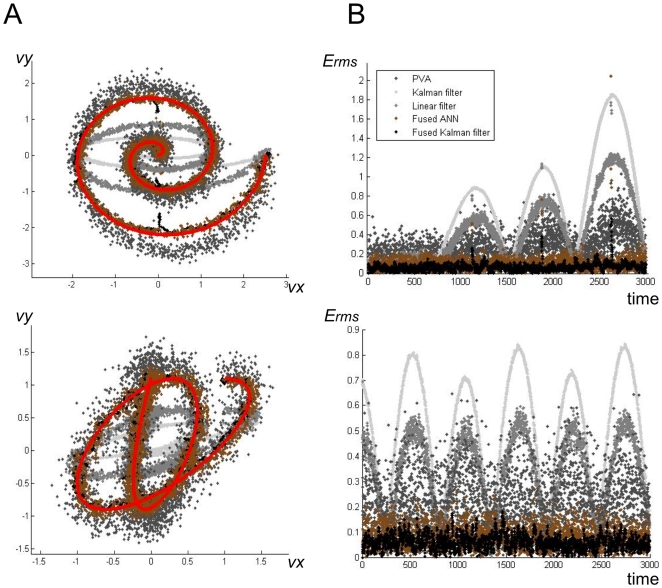
Fusion results of using potentially poor quality decoders. These two sets correspond to trials 2 and 3 in Table 3. (A) Example trials showing individual and fusion decodes. True velocities are shown in red. The fused ANN and fused Kalman filter decodes are shown in brown and black, respectively. Individual decoders are plotted in varying shades of grey. (B) Corresponding pointwise root mean squared error of decodes over time. Note that time is unitless in these simulations. Though the decoders have variable accuracy over time, the fusion algorithms maintain acceptable decoding accuracy throughout the entire trials.

To address this scenario, we subsequently tested the ability of our fusion algorithms to handle poor quality decoding. Generating a simulated neural training set lacking sufficient complexity and size, we retrained the individual decoders resulting in unacceptable decoding accuracy. We ran four decoding trials, comparing the fusion outputs to the single decoders. In Figure 4 and **Table 3**, we observe the poor performance of the Kalman filter and optimal linear filter decoders. Despite the high error associated with each single decoder, the fusion algorithms successfully produce highly accurate decodes, significantly improving over all three individual decoders. Note that we again optimized the ANN topologies for each trial similarly to the previous experiment. In Figure 4B, we analyzed the decoding accuracy of each algorithm over time for trials 2 and 3. While the error for the individual decoders varies over time, the fusion algorithms effectively assessed the individual decoders' weaknesses, and resulted in lower *E_rms_* throughout the entire trials.

**Table 3:** *E_rms_* ± standard error (s.e.) for four trials with variable decoding accuracies.

Trial	1	2	3	4
**Kalman filter**	0.557±0.009	0.828±0.017	0.549±0.009	0.898±0.017
**Linear filter**	0.371±0.006	0.550±0.012	0.365±0.006	0.523±0.010
**Population vector**	0.248±0.005	0.318±0.013	0.238±0.004	0.285±0.005
**Kalman fusion**	0.108±0.002	**0.079±0.012**	**0.063±0.001**	0.198±0.003
**ANN fusion**	**0.098±0.002**	0.116±0.005	0.091±0.002	**0.121±0.002**

Bold elements in tables have the lowest *E_rms_* for the trial. In all four trials, the fusion algorithms had more accurate results than **all** three individual decoders. Trials 2 and 3 are displayed in Figure 4.

**Figure 5:**
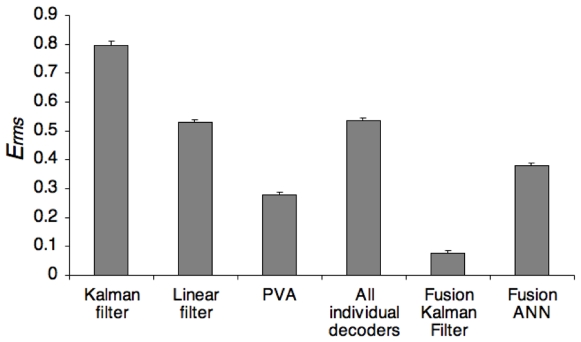
Results of decoders on 468 random trajectories (E_rms_ mean ± s.e.). The improvement of fusion algorithms over the combined individual decoders was statistically significant (**p<1e-29** in both cases, two-tailed Welch's T-test). While the fusion Kalman filter produced the significantly more accurate outputs than the individual decoders, the ANN limited to a single topology did not perform as well, illustrating an advantage of the Kalman filter as a fusion method.

To determine if the improvement of the fusion algorithms was statistically significant, we generated 468 additional randomized trajectories (selected from a large space of smooth realistic movements, see Methods) and corresponding simulated neural spike datasets. For each trial, we employed only a single ANN topology, because searching a space of topologies is not feasible for real-time decoding. The selected ANN used a single hidden-layer with six hidden units, the same as the number of input nodes. The fusion Kalman filter resulted in significantly lower *E_rms_* than all three individual decoders, (***p*** **<1e-150** in all cases, one-tailed paired T-test) (see Figure 5). The ANN fusion method was not as successful, though still produced significantly more accurate decodes than the Kalman filter and linear filter single decoders, (***p*** **<1e-44** for both comparison, one-tailed paired T-test). Our PVA variant resulted in significantly more accurate decodes than ANN fusion (***p*** **<1e-42**, one-tailed paired T-test). Since it is not reasonable to find an optimal ANN topology in real time, the Kalman filter has a major advantage over the ANN as a fusion method. However, if a topology could be found in training that performed well overall, then the ANN would provide a computationally efficient method for decision fusion.

## Discussion

We have described a framework for fusing decisions in the context of multimodal prosthetic devices. Investigating the Kalman filter and ANNs, we have shown that each fusion method is capable of producing accurate fusion decodes and can adapt to decodes of varying quality over time.

While our expertise is targeted towards neural decoding for prosthetic limb movement, this approach may be generalized to the larger field of brain-machine interfaces (BMIs) to help improve communication for patients suffering from severe paralysis, locked-in syndrome, and other neurological injuries. Recent BMI studies have demonstrated success in providing some level of communication for subjects [41], [45], though to our knowledge, none have employed a fusion framework for decoding. As hardware platforms for neural recording continue to advance, so too will our opportunities for fusing multiple signals with distinct characteristics.

The computational expense of a fusion step in a neural prosthetic device is of notable importance. Each of the methods examined in this study is capable of running in real-time on a single processor, which is likely to be the hardware implementation of such a framework. Furthermore, the computational cost of individual modality decoders is increasing considerably, with many suggesting parallel processing implementations [46], [47]. The efficiency of these fusion algorithms could be improved by reducing the dimensionality of the data using feature selection or principal component analysis [22].

Progress in neural recording technologies may eventually lead to opportunities for data fusion, where a single decoder is used on all modalities simultaneously. Our choice to employ decision fusion in this study was in large part due to the current capabilities of neural prostheses and those in development, making our findings timely.

Our results must be qualified because of the artificial nature of our cortical spike data. Though our analysis is based on simulated neural activity, we sought to capture the fundamental features of spike data including: a realistic number of monitored neurons, randomized preferred directions, and firing rates exhibiting Poisson noise. Our simulated neurons are indeed close to ideal, but we have shown the significant improvement decision fusion can provide when fusing predictions from decoders of variable accuracy – a result independent of the simulated data itself. Currently, no continuous real-time multimodal neural data recordings are available, but several are in production, and the community has shown an evident interest in this direction [48], [49]. We plan to perform a rigorous off-line evaluation of decision fusion and data fusion methodologies using real multimodal neural data in future work.

An ideal neural prosthesis will be fully autonomous, capable of independently retraining and adapting to different human conditions and mechanical failure. Electrode loss is arguably the most important limiting factor for neural prostheses proliferation [17], [18], [19], and multiple craniotomies are not a practical solution. As a corollary, an autonomous prosthetic arm will need to detect recording anomalies and adjust appropriately. If individual decoders do not address this issue, any fusion technique is susceptible to electrode loss. However, some fusion methods are easily modified to adapt to this problem. The Kalman filter and other methods may be formulated such that poor quality decoders can be isolated and removed from the prediction without retraining, while the ANNs would be significantly more problematic. We hope to extend these methods to provide better autonomy in the future.

Neural prosthetics is a swiftly evolving field with ambitious goals. Restoring the functionality of a limb for an individual will require innovative technology and robust computational methods to rapidly and accurately assess user intent.
